# Evaluating Interpersonal Synchrony: Wavelet Transform Toward an Unstructured Conversation

**DOI:** 10.3389/fpsyg.2016.00516

**Published:** 2016-04-12

**Authors:** Ken Fujiwara, Ikuo Daibo

**Affiliations:** ^1^Faculty of Human Sciences, Osaka University of EconomicsOsaka, Japan; ^2^School of Motivation and Behavioral Sciences, Tokyo Future UniversityTokyo, Japan

**Keywords:** non-verbal behavior, interpersonal coordination, synchrony, spectrum analysis, wavelet transform, an automated method

## Abstract

This study examined whether interpersonal synchrony could be extracted using spectrum analysis (i.e., wavelet transform) in an unstructured conversation. Sixty-two female undergraduates were randomly paired and they engaged in a 6-min unstructured conversation. Interpersonal synchrony was evaluated by calculating the cross-wavelet coherence of the time-series movement data, extracted using a video-image analysis software. The existence of synchrony was tested using a pseudo-synchrony paradigm. In addition, the frequency at which the synchrony occurred and the distribution of the relative phase was explored. The results showed that the value of cross-wavelet coherence was higher in the experimental participant pairs than in the pseudo pairs. Further, the coherence value was higher in the frequency band under 0.5 Hz. These results support the validity of evaluating interpersonal synchron Behavioral mimicry and interpersonal syyby using wavelet transform even in an unstructured conversation. However, the role of relative phase was not clear; there was no significant difference between each relative-phase region. The theoretical contribution of these findings to the area of interpersonal coordination is discussed.

## Introduction

Interpersonal coordination has attracted the attention of social psychology and communication researchers. Past work has revealed synchronization or unsynchronization at various levels of communication behavior; for instance, vocal intensity (Natale, 1975), vocalization duration (Cappella and Planalp, 1981), speech rate and response latency (Street, 1984), eye movements (Richardson and Dale, 2005), body posture (Scheflen, 1964), and body posture sway (Shockley et al., 2003) are coordinated between interactants. Communication Accommodation Theory (Giles et al., 1991) and Interpersonal Adaptation Theory (Burgoon et al., 1995) provide theoretical frameworks to explain how conversational features, including vocal patterns or gestures, become synchronized or unsynchronized between conversation partners.

Previous studies have suggested that there is a link between coordination and pro-sociality. Coordination leads to rapport (Tickle-Degnen and Rosenthal, 1990), affiliation (Hove and Risen, 2009), and cooperation (Wiltermuth and Heath, 2009) between interactants. One study found that the experience of coordination resulted in voting for left-wing parties, which is considered a prosocial behavior (Stel and Harinck, 2011). Conversely, coordination also occurred as a result of pro-sociality. The goal to affiliate or create rapport increases coordination (Lakin and Chartrand, 2003). Similarly, pro-social orientation increases the propensity to coordinate with others (Lumsden et al., 2012b). Many studies show consistent findings regarding the relationship between coordination and pro-sociality or positive interpersonal relationships; however, the definition of coordination is a bit more complex. There are large variations in how to study and analyze coordination.

### Interpersonal Coordination in Time- or Frequency-Domain

**FIGURE 1:**
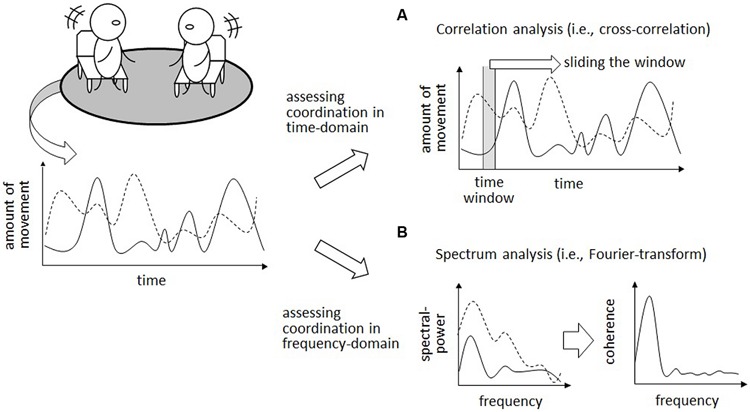
**Example of interpersonal coordination in time- and frequency- domains.** This example illustrates the coordination of the amount of movement between two interactants in the time- **(A)** and frequency- **(B)** domains.

Interpersonal coordination, by definition, occurs when two or more individuals coordinate their behavior in a time series. The relationship between each time series can be analyzed in either time- or frequency-domains (Issartel et al., 2006). Therefore, coordination can be considered as a time- and/or frequency-domain phenomenon. In the time-domain, the amount of movement or the occurrence of a specific behavior is plotted on the *y*-axis while the *x*-axis represents the timeline. Coordination is interpreted as the extent to which behaviors co-occur or the amount of behavior that is similar between the interactants within a predetermined time window (**Figure 1**). In comparison, in the frequency-domain, the extent of spectrum power is plotted on the *y*-axis while the *x*-axis represents frequency components. Coordination, in this case, is represented as the amount of similarity at each frequency component (i.e., cross-spectral coherence). Each instance of time- or frequency-domain coordination seems to correspond approximately to the work of Bernieri and Rosenthal (1991) who differentiated interpersonal coordination into two facets: behavior matching and synchrony.

Behavior matching, in the early stage of research, was defined as the similarity of body postures between interactants. Researchers focused on whether their posture was congruent in a predetermined time window throughout the time series. Postural congruence has been observed in interactants who share a common viewpoint (Scheflen, 1964) and found to lead to rapport (LaFrance, 1976, 1979). Although behavioral ratings have been employed to assess postural congruence between interactants (e.g., Bernieri, 1988), some studies have suggested possible bias in the rating of raters (Cappella, 1990; Lumsden et al., 2012a). Therefore, recent studies have directly assessed postural congruence using various sensors or motion-capture technologies (Poppe et al., 2014; Won et al., 2014), which enable objective measurements of the similarity of postures throughout the time series.

In the recent literature, behavioral matching is known and described as behavioral mimicry (e.g., Chartrand and Bargh, 1999; Hove and Risen, 2009; Lakin, 2013). Behavioral mimicry is an automatic tendency to imitate another’s behavior at a particular moment in time. The target of behavioral mimicry is broad; it includes posture as well as gestures, mannerisms, and other motor movements (for a review see Chartrand and Lakin, 2013; Lakin, 2013). Behavioral mimicry is typically assessed by examining whether the same or a similar behavior occurs at a given point in time or whether the presented behavior is mimicked by an interactional partner within a short window of time. For instance, Lakin and Chartrand (2003) measured the frequency of similar or identical behaviors (i.e., face touching) between participants and the confederates in experimental tasks. Tschacher et al. (2014) measured the similarity of motor movement between interactants in a dyadic face-to-face situation. The extent of the similarity was assessed by employing time-lagged cross-correlations using two different time windows: one was a 10-s window for the computation of cross-correlations, and the other was 30 s that was used to account for non-stationarity. Although the window size and target behaviors were different in each study or its purpose, behavioral mimicry research has shed light on coordination in the time domain.

Over time, synchrony research has focused on the similarity of rhythm and timing, which can be interpreted as a frequency-domain phenomenon. Synchrony research employing an analysis method from physics (e.g., Pikovsky et al., 2003) has revealed that temporal coordination occurs between interactants (e.g., Schmidt and O’Brien, 1997), and that it can increase affiliation (Hove and Risen, 2009). Some synchrony research has focused on similar movements between interactants such as swinging pendulums and rocking in rocking chairs (e.g., Schmidt and O’Brien, 1997; Richardson et al., 2007); however, synchrony can be achieved even with different behaviors between interactants. Bernieri and Rosenthal (1991), for instance, suggested jazz as an example of synchrony. Jazz players each have an instrument and play it in a different manner from other players as the mechanism of playing a guitar differs from that of a saxophone. The essence of synchrony is rhythm and timing. In order to evaluate synchrony in the frequency domain (i.e., the similarity of rhythm and timing), a simple correlation applied to assess time-domain coordination cannot be employed. Spectrum analysis is used in such situations.

### Evaluating Synchrony: Spectrum Analysis

To evaluate synchrony in the frequency domain, a spectrum analysis that deconstructs a complex time-series into its rhythmic components, was employed (e.g., Schmidt and O’Brien, 1997; Schmidt et al., 2012). Spectrum analysis is applied to time-series continuous data that refer to scales in which the interval between observations (i.e., sampling rate) is constant; nominal and ordinal data cannot be used for spectrum analysis (Issartel et al., 2014).

In the early year of non-verbal research, microanalysis (e.g., Condon and Ogston, 1966, 1967) was used to analyze films of social interactions, frame by frame to generate time-series movement data. This measuring process, unfortunately, tends to be resource intensive. Coding behaviors is time-consuming and painstaking in itself and requires establishing reliability among the coders. It is not unusual to sped twice the length of a film’s time coding the behavior of interest. To address this problem, some recent studies have utilized automatic techniques to generate time-series movement data; they use the depth sensor, Kinect (Microsoft; Frauendorfer et al., 2014; Won et al., 2014), or employ video-tracking techniques (Kupper et al., 2010; Schmidt et al., 2012; Paxton and Dale, 2013; Fujiwara and Daibo, 2014; Tschacher et al., 2014). Behavioral data acquired using these techniques can be less costly and highly reliable.

After obtaining time-series data, a spectrum analysis can be conducted. The Fourier transform is one of the well-known types of spectrum analysis. It calculates a spectral power that indicates the magnitude at each component frequency. If there are two time-series, cross-spectrum analysis can be applied to them and coherence can be calculated. Coherence, which ranges on a scale of 0–1, is a measure of similarity between the two time-series at each component frequency. A coherence of 1 reflects a perfect correlation between the two movements, and 0 reflects no correlation (Schmidt and O’Brien, 1997; Richardson et al., 2007). Schmidt et al. (2012) conducted a periodic interaction task (i.e., telling knock–knock joke) and applied the Fourier transform toward the time-series movement data of the interactants. The mean value of coherence at the dominant rhythm, large spectral peaks at specific frequencies (i.e., 0.125 and 0.5 Hz), was calculated to evaluate rhythmic similarity.

Spectrum analysis, including the Fourier transform, also provides phase information. In synchrony research, relative phase angle, which indicates a time lag at the frequency between interactants, has been used (e.g., Schmidt et al., 2012). More precisely, Schmidt et al. (2012) used the Hilbert transform, a filter that simply shifts the phases of all frequency components of its input by -π/2 radians, to conduct the relative phase analysis. A 0° relative phase indicates movements in the same part of their cycles at a given time, which is called *in-phase* patterning. On the opposite end of the range, a 180° relative phase indicates movements in the opposite parts of their cycles at a given time, which is called *anti-phase* patterning. In the periodic interaction task, Schmidt et al. (2012) demonstrated the robustness of relative phase analysis across multiple measures and identified significant periods of coordination. Relative phase information, combined with coherence value, is used as a tool for analysis to explore the dynamic synchronization process of the interactants.

However, the Fourier transform that has been used in previous studies has a serious practical limitation: it assumes a stable frequency or repetitive pattern during the entire interaction (Issartel et al., 2006). The use of periodic or rhythmic tasks makes it easier to control turn-taking between the interactants and enables the capture of the dominant rhythm. If a rhythmic task requires a specific behavior once every 8 s, a large spectral peak will be found at approximately 0.125 Hz, which means that the dominant rhythm in the situation is 0.125 Hz. Interactants engaging in the same rhythmic task usually have a dominant rhythm with each other, and researchers can evaluate the extent of rhythmic synchronization by analyzing the degree of similarity at the dominant rhythm. On the contrary, in our daily conversations (i.e., unstructured conversations), it is difficult to assume a stable frequency and a repetitive pattern of movements, which means that the dominant rhythm cannot be set prior to a conversation. Even during one’s turn, some parts may become faster and others slower. For interactants, their movements may be synchronized in a faster rhythm at some points and in a slower rhythm at others. Therefore, the Fourier transform does not appear to be the best way to evaluate synchrony in the frequency domain in an unstructured conversation.

### Wavelet Transform: Coordination in Time–Frequency Plane

**FIGURE 2:**
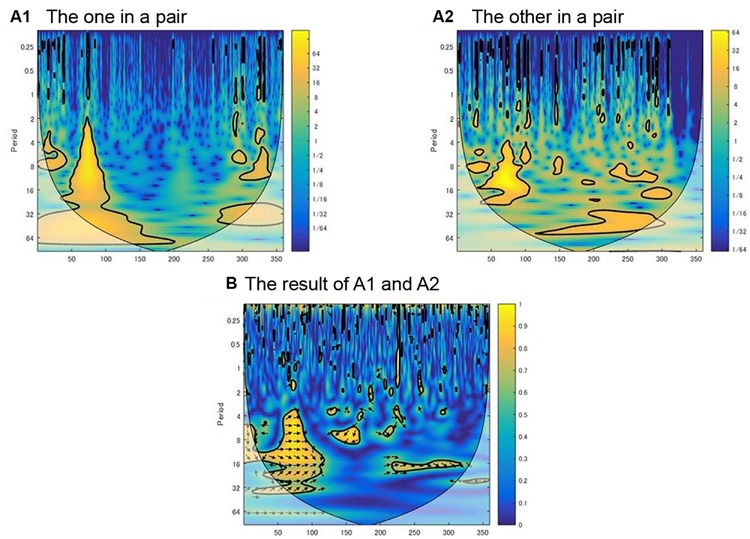
**Example of interpersonal coordination in time-frequency plane.** Two images of wavelet power **(A1,A2)** and one of the cross-wavelet coherence **(B)** of A1 and A2 are shown. The time line is represented on the *x*-axis (360 s) and each frequency component is represented on the *y*-axis, as an inverted period (e.g., 0.25 period is 4 Hz). The magnitude of wavelet power and wavelet coherence is represented by color. **(B)**, The relative phase at a given frequency and time point is denoted by the orientation of the arrow: the right arrow indicates *in-phase* synchronization, the left arrow indicates *anti-phase* synchronization, and the downward arrow indicates no synchronization. The average value of these variables at a given frequency was extracted from these plots to analyze the coherence and relative phase. However, the cone of influence (COI) area that is shown as a lighter shade is not included in the analysis.

The wavelet transform can be a potent alternative to the Fourier transform. It does not require stationarity in each time series. By employing the wavelet transform, time-series movement data is plotted onto a time–frequency plane (**Figure 2**). In the time–frequency plane, the frequency components are illustrated on the *y*-axis while the *x*-axis represents the time line, and the spectrum power is represented by the gray value, which illustrates the extent of spectrum power, which changes throughout the time line. As with the Fourier transform, cross-spectrum analysis can be conducted using the wavelet transform, and cross-wavelet coherence represents the similarity between the two time series at each component frequency throughout the time line.

Several studies of interpersonal movement coordination have evaluated coordination by using cross-wavelet analysis. Walton et al. (2015) demonstrated that the cross-wavelet approach could illustrate the dynamics of movement coordination between improvising musicians. Varlet et al. (2011) reported that a pair of participants engaged in a visual tracking task influenced one another, and produced spontaneous postural coordination. Varlet et al. (2011) also used phase information via cross-wavelet transform to evaluate the occurrence of postural coordination. In a study of cross-wavelet coherence, Washburn et al. (2014) collected body movement data in dance settings, and found that the cross-wavelet coherence of the trained dancers was significantly higher than that of the non-dancers, indicating that the dancers achieved a higher level of coordination with their confederate. Sofianidis et al. (2012) performed a rhythmical sway task in the sagittal plane, and found that a light fingertip contact, i.e., haptic contact, increased coherence. In settings with more socialization, Schmidt et al. (2014), as well as Schmidt et al. (2012), used the knock–knock joking task, and calculated wavelet coherence to evaluate rhythmic similarity between two interactants. Schmidt et al. (2014) revealed that the bodily synchronization in the joke-telling task occurred at the dominant rhythms as well as across different nested temporal scales. Relative phase information also indicated that in-phase synchronization rather than anti-phase synchronization was observed between the interactants, which supported and validated the findings of Schmidt et al. (2012).

### Current Study

Many previous studies employing wavelet transform were conducted under situations that were a less social (e.g., Varlet et al., 2011) or involved a specific task (Schmidt et al., 2014). This study did not employ a specific rhythmic task but rather focused on an unstructured conversation. In this type of situation, the dominant rhythm could be determined prior to the conversation because conversation speed is not predictable. Additionally, turn-taking between the interactants was not controlled. We examined whether the coordination represented in the time-frequency plane would be observed even in an unstructured conversation.

The study’s setting was based on the previous studies in social interaction research (e.g., Bernieri, 1988; Bernieri et al., 1988; Chartrand and Bargh, 1999; Lakin and Chartrand, 2003; Tschacher et al., 2014); thus, our participants were seated to engage their conversation. Compared to interpersonal movement coordination studies (e.g., Varlet et al., 2011; Washburn et al., 2014; Walton et al., 2015), these settings minimized participants’ movements, which required us to conduct a conservative test. However, we addressed this issue by focusing on typical kinesics indicators: hand and head movements. Hand movements, including gestures (Ekman and Friesen, 1969, 1972) and/or head movements, including nodding (Hadar et al., 1985; Frauendorfer et al., 2014), could be seen even if participants were seated. Moreover, previous studies revealed that hand and/or head movements are coordinated in face-to-face interactions (Holler and Wilkin, 2011). Even if the interactants were seated, it would be possible to examine whether their movements were synchronized by extracting the head and hand movements.

To test the existence of synchrony, Bernieri and Rosenthal (1991) proposed the pseudo-synchrony experimental paradigm. In this paradigm, video clips of dyadic interaction partners (i.e., a genuine pair) are isolated and re-combined in a random order. The synchrony scores of these virtual data (i.e., pseudo pairs) are compared to the genuine pair. Employing the wavelet transform, we hypothesized that the extent of synchrony, as represented by the cross-wavelet coherence coefficient, would be higher in the genuine pairs than in the pseudo pairs. In addition, we explored whether there’s a difference in the relative phase (i.e., in-phase and anti-phase) in the genuine pairs.

## Materials and Methods

### Participants

Seventy-four Japanese female undergraduates participated in exchange for extra course credit. Each participant was randomly paired with a stranger. The familiarity between the participants was expected to have a potent influence on the strength of synchrony; therefore, after their conversation, the participants were asked to complete questionnaires regarding their familiarity with one another. Five pairs who knew each other were removed from the subsequent analysis. In one case, the conversation was not recorded due to a malfunction of the video equipment. Therefore, a total of 31 dyads from 62 participants (Mean age = 18.47, *SD* = 0.59) were analyzed.

### Procedures

First, participants were seated back-to-back and completed a consent form; then they moved to another seat where they were positioned opposite one other, 80 cm apart. They were instructed to engage in a 6-min conversation and become acquainted. The conversation topics were not specified, and the participants did not know the conversation would be analyzed from the perspective of synchrony. Their conversation was video-recorded using a camera (HDR-SR12; SONY) placed at a distance of 250 cm, and to the right side of the participants.

### Ethics Statement

All procedures performed in studies involving human participants were in accordance with the ethical standards of the Department of Human Sciences in Osaka University.

### Generating Time-Series Movement Data

There are several methods of generating time-series movement data; some use a depth sensor (Frauendorfer et al., 2014; Won et al., 2014), and others employ video-tracking techniques (Kupper et al., 2010; Schmidt et al., 2012; Paxton and Dale, 2013; Tschacher et al., 2014). In this study, time-series movement data was extracted using video-images analysis software (Dipp-MotionPRO Ver. 2.24c). By using three attributes of color, this software automatically tracks and captures two-dimensional body movements in chronological order. Previous research (Fujiwara and Daibo, 2014), using a former version of this software (Dipp-Motion XD Ver. 3.20-2), demonstrated that gestures categorized by information on a coordinate point corresponded closely with a third person’s judgment (Spearman rank correlations: rs = 0.78). This finding indicates that this software can track and capture body movements with high resolution, even if the movement is not very large.

For each participant, coordinate points for the fingertips and nose were captured in chronological order. Time resolution was set to 0.1 s. After down-sampling, the number of coordinate point changes between the adjacent video frames was calculated for the movement of each body part (i.e., fingertips and nose). The movement data from each video was calibrated using the size of an outlet cover (7 cm × 12 cm). After that, the movements of the fingertips and nose were added together.

### Generating Virtual Data

To evaluate the significance of the extent of synchrony in the genuine pair, a baseline was needed. Based on the pseudo-synchrony experimental paradigm (Bernieri and Rosenthal, 1991), a virtual dataset was generated. Data from two time-series from the genuine pair were isolated and re-combined in random order. This shuﬄing procedure can keep the structure of the original movement intact, and thereby yield a statistically more conservative test (Ramseyer and Tschacher, 2010). The extent of synchrony of the pseudo pairs was assessed in the same manner.

### Evaluating Synchrony with Wavelet Coherence

By using Matlab 2014a (Mathworks) and the wavelet toolbox (Grinsted et al., 2004), we conducted a wavelet transform for each time series. The default parameters of Grinsted et al. (2004) were employed except for the number of the order; following Issartel et al. (2006), the order was set to eight. Morlet was used as the mother wavelet. To evaluate the rhythmic similarity between two individuals, cross wavelet coherence was calculated. The cone of influence (COI) area was not included in subsequent analyses (Grinsted et al., 2004). We used a coherence value under 4 Hz because our participants’ unstructured conversation with a stranger was not active or fast. The average coherence under 4 Hz across the time line, was standardized by using a Fisher-Z transformation before the statistical analyses. In addition, to determine which frequency band was sensitive enough to illustrate synchrony in the genuine pair, the average coherence of each frequency band (under 0.025, 0.025–0.05, 0.05–0.1, 0.1–0.2, 0.2–0.5, 0.5–1, 1–2, 2–3, and 3–4 Hz, respectively) was calculated and compared to the others.

Additionally, in the genuine pairs, the relative phase in nine 20° regions from 0 to 180° was extracted. Because dominant rhythm cannot be predetermined in an unstructured conversation, it was not clear which frequency to focused on in order to extract the relative phase. Therefore, in the area where wavelet coherence was significant, the number of occurrences in each 20° region was counted and the percentage distribution was calculated for each pair. The proportion of each region was transformed via arcsine transformation, which was used in the subsequent analysis.

## Results

We compared the coherence values between the genuine pairs and the pseudo pairs. As anticipated, the result of separate *t*-tests indicated that the average coherence under 4 Hz throughout the time line was higher in the genuine pairs (*M* = 0.26, *SD* = 0.02) than in the pseudo pairs (*M* = 0.24, *SD* = 0.02), and this difference was significant [*t*(59.52) = 2.22, *p* = 0.030, *d* = 0.56].

In addition, the coherence value in the genuine pairs was submitted to a one-way ANOVA with a within-subjects variable of frequency band (under 0.025, 0.025–0.05, 0.05–0.1, 0.1–0.2, 0.2–0.5, 0.5–1, 1–2, 2–3, and 3–4 Hz). The result indicated that the main effect of frequency band was significant [*F*(8,240) = 11.73, *p* < 0.001, $\eta_{p}^{2}$ = 0.28]. Holm’s multiple comparison revealed that 0.5 Hz was the boundary of the extent of synchrony. The coherence value decreased as the frequency increased, and there was a significant difference between coherence at 0.2–0.5 and 0.5–1 Hz (**Table 1**).

**Table 1:** Means and standard deviations of wavelet coherence for each frequency component.

<0.025 Hz	0.025–0.05 Hz	0.05–0.1 Hz	0.1–0.2 Hz	0.2–0.5 Hz	0.5–1 Hz	1–2 Hz	2–3 Hz	3–4 Hz
0.330_a_	0.302_a_	0.288_a_	0.270_ab_	0.248_b_	0.232_c_	0.231_c_	0.228_c_	0.226_c_
(0.135)	(0.099)	(0.068)	(0.044)	(0.022)	(0.023)	(0.014)	(0.013)	(0.013)

*The standard deviations are in parentheses below the means; the means with different subscripts are significantly different based on Holm’s multiple comparison (adjusted p-value < 0.05).*

**FIGURE 3:**
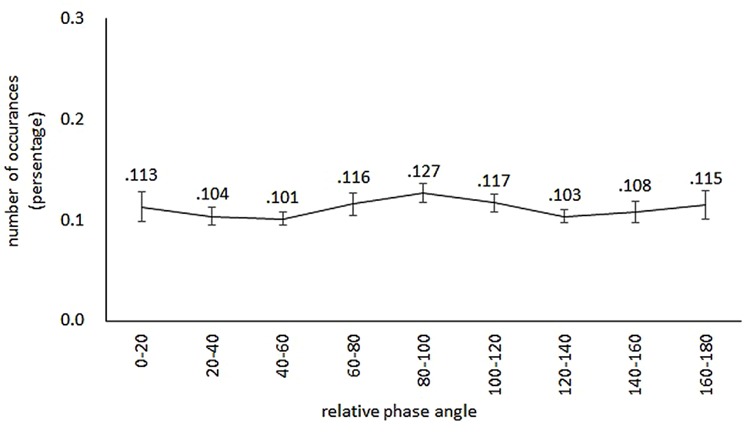
**Percentage distribution of relative phase occurrences.** The mean value is indicated above the error bar representing the Standard Error. Relative phase is distributed in nine 20° regions from 0 to 180°. The distribution is almost equally spread over the nine regions of the relative phase angle.

The proportion of relative phase in the genuine pairs was submitted to a one-way ANOVA with a within-subjects variable of relative phase angle (each 20° region from 0 to 180°). The result indicated that the main effect of relative phase angle was not significant [*F*(8,240) = 0.57, *p* = 0.802, $\eta_{p}^{2}$ = 0.02; **Figure 3**].

## Discussion

This study examined whether the coordination represented in a time–frequency plane could be seen in an unstructured conversation. The results of employing the wavelet transform and calculating the wavelet coherence indicated that the genuine pair who had a conversation was more synchronized than the pseudo pair consisting of virtual data, which supported our hypothesis and the validity and possible utility of the cross-wavelet approach.

Findings of the current study extend the field of synchrony research. Interpersonal coordination was observed at various levels of communication behavior (Giles et al., 1991; Burgoon et al., 1995, for a review). Bernieri and Rosenthal (1991) illustrated two aspects of interpersonal coordination: behavioral matching and synchrony, which can be considered time- and frequency-domain phenomena. Behavioral matching or behavioral mimicry have been examined in studies using various situations (e.g., Scheflen, 1964; LaFrance, 1976, 1979; Chartrand and Bargh, 1999; Lakin and Chartrand, 2003). However, compared to behavioral mimicry research, the findings of synchrony research are limited. Many previous studies were conducted under a specific task situation (e.g., Schmidt et al., 2012) or less social situations (e.g., Varlet et al., 2011). This study employed a spectrum analysis using the wavelet transform and found that synchrony, in this case rhythmic similarity, was observed even in an unstructured conversation; there were no rhythmic tasks or restrictions on turn-taking. Moreover, as the current data were tested in a conservative fashion, the results are not very strong but their significance show the robustness of the cross-wavelet analysis. Our study contributes to the literature by extending the usage of cross-wavelet approach to a social interaction situation, and adds new insight regarding rhythm to communication research focusing on daily conversations.

In our daily conversations, the dominant rhythm is not predetermined. However, our findings suggest that the interactants achieved synchrony even if they were not engaged in a specific rhythmic task, which can be captured by using the wavelet transform. These findings seem to generate new questions about the frequency at which people actually synchronize, and at which temporal synchronization people perceive as being comfortable and smooth. The findings of this study provide a clue to address the first question. In the genuine pair, the value of wavelet coherence was significantly higher under 0.5 Hz. This frequency was seen in a previous study that employed a periodic rhythmic task (e.g., Schmidt et al., 2012). Although employing a specific task makes it possible to achieve synchronization at a faster rhythm (about 1.33 Hz; Schmidt et al., 2014), our daily conversations might not move so quickly. Our findings are not sufficient to illustrate the temporal characteristics of our face-to-face interactions. More empirical data should be collected. In future research, it would be important to explore the antecedents of tempo when people achieve synchronization. Whether the specific (e.g., fast or slow) temporal synchronization can be perceived as comfortable and/or smooth by interactants remains to be explored.

In addition, the role of relative phase should be pursued. In the current study, the characteristics of relative phase were not clear; there was no significant difference between in-phase and anti-phase synchronization. Although previous studies revealed that interactants synchronized in the in-phase (e.g., Schmidt et al., 2012) and that in-phase synchronization had a positive relationship with affiliation (Hove and Risen, 2009), these findings were from rhythmic task situations. If in-phase and/or anti-phase synchronization is achieved even in an unstructured conversation, which factors make this possible? Behavioral mimicry research has indicated that a positive attitude or an intention of affiliation toward one’s partner causes behavioral mimicry (e.g., Lakin and Chartrand, 2003). Similarly, positive attitudes or intentions of affiliation toward one’s partner might influence on in-phase and/or anti-phase synchronization. In addition to the antecedents, the results of in-phase and/or anti-phase synchronization need to be examined. In-phase synchronization is a perfect match in rhythm and timing, whereas anti-phase synchronization is a matching of rhythm, but not timing. Although a previous study showed that both types of synchronization had a positive influence on third-party judgments of rapport (Miles et al., 2009), it is not yet known whether the difference in timing had a different influence on the actual interactants, which remains a question for future research.

### Limitations and Directions for Future Research

The limitations of this study include the lack of a measure of rapport. We corroborated the existence of synchrony by using the wavelet transform and pseudo synchrony paradigm (Bernieri and Rosenthal, 1991); the genuine pairs of interactants were more synchronized than the pseudo pairs. Interpersonal coordination studies, including behavioral mimicry and synchrony research, have revealed a positive relationship between coordination and rapport (e.g., LaFrance, 1976, 1979; Tickle-Degnen and Rosenthal, 1990; Hove and Risen, 2009). If wavelet transform is employed, there should be several questions regarding the relationship with rapport; for instance, whether, and at which frequency, wavelet coherence is related to rapport, and whether in-phase and anti-phase synchronization are related to rapport. Our findings support the validity and possible utility of wavelet transform to evaluate the extent of synchrony in our daily conversations. Thus, to develop synchrony research, further examination using the wavelet transform is needed.

Although the current study has a limitation, wavelet transform seems to have the potential to contribute to the theoretical development of interpersonal coordination. Bernieri and Rosenthal (1991) differentiated two facets of interpersonal coordination: behavior matching (or behavioral mimicry) and synchrony. Although the difference between the two facets is still argued (Chartrand and Lakin, 2013; Lakin, 2013), researchers have not yet reached a clear conclusion. This is partly because of the lack of methodology used to differentiate and integrate behavioral matching (or mimicry) and synchrony. To this end, the wavelet transform can be a powerful analysis method. The coordination assessed in the time-frequency plane (i.e., wavelet transform) indicates the extent of the rhythmic similarity located in the time line. Using the time line, the boundary between behavioral mimicry and synchrony would become blurred, and the difference between them could be regarded as a difference in perspective about coordination, not as different phenomena. Synchrony in the time-frequency plane represents how similar the rhythm or velocity between the interactants is, across the time line. In contrast, behavioral mimicry in the time-domain represents the extent to which behaviors co-occur or how similar the amount of movement is across the time line. In this perspective, synchrony and behavioral mimicry are distinguished by their focus, with the former focusing on velocity, and the latter focusing on the amount. Furthermore, the similarity of synchrony and behavioral mimicry can be argued because researchers conduct spectrum analysis and cross-correlation analysis on the same data (i.e., two movement time-series). Differentiating and integrating synchrony and behavioral mimicry should facilitate the development of coordination theory.

## Author Contributions

All authors contributed to the study design. KF performed the data collection, analysis, and interpretation. KF drafted the manuscript, and ID provided critical revisions. All authors approved the final version of the manuscript for submission.

## Conflict of Interest Statement

The authors declare that the research was conducted in the absence of any commercial or financial relationships that could be construed as a potential conflict of interest.
